# Empowering rural physicians through an AI-assisted intervention for deprescribing in older adults with comorbid diabetes and hypertension: protocol for a feasibility study

**DOI:** 10.3389/fmed.2026.1906806

**Published:** 2026-10-01

**Authors:** Yu Xia, Ming Yan, Leyi Jiang, Yang Zhao, Yang Li, Mi Yao, Ruoxia Zhang, Junhai Zhen, Xinhui Yang, Lingyan Wu, Yi Guo, Zhijie Xu

**Affiliations:** 1 Department of General Practice, Peking University Shenzhen Hospital, Shenzhen, China; 2 Department of Emergency Medicine, Sir Run Run Shaw Hospital, Zhejiang University School of Medicine, Hangzhou, China; 3 Department of General Practice, the Second Affiliated Hospital, Zhejiang University School of Medicine, Hangzhou, Zhejiang, China; 4 Duke-NUS Medical School, National University of Singapore, Singapore, Singapore; 5 Department of General Practice, Shanghai East Hospital, Tongji University School of Medicine, Shanghai, China; 6 Department of General Practice, Peking University First Hospital, Beijing, China

**Keywords:** artificial intelligence agent, comorbidity, deprescribing, diabetes, feasibility study, hypertension, study protocol

## Abstract

**Background:**

In China's primary healthcare system, older adults with both hypertension and diabetes are a key focus. These patients often deal with polypharmacy, which heightens the risk of adverse drug reactions, lowers treatment adherence, and increases medical burdens. Deprescribing, an evidence-based approach to optimizing medication regimens, is challenging for rural physicians due to barriers like limited clinical knowledge, lack of systematic decision support, and time constraints. Artificial Intelligence (AI) presents a promising solution, yet the feasibility of AI-assisted deprescribing in rural primary care is underexplored. This protocol describes a study designed to evaluate the feasibility, acceptability, and appropriateness of an AI agent for rural physicians managing older adults with these comorbidities.

**Methods and analysis:**

This protocol outlines a single-arm, pre-post feasibility study to be conducted in rural primary healthcare institutions across 11 administrative regions in Zhejiang Province, China. The study aims to recruit 30 eligible rural physicians and at least 150 older patients (aged ≥65 years) who have concurrent diagnoses of hypertension and diabetes and are taking five or more long-term medications. The intervention utilizes an AI agent with clinical medication guidance functions to assist rural physicians in the deprescribing process. The follow-up period spans 6 months. The primary outcome is the change in the total number of medications per patient after the intervention. Secondary outcomes include patient clinical outcomes and rural physicians' acceptance of the AI agent. Quantitative data will be analyzed using descriptive and inferential statistics, while qualitative data from semi-structured interviews will undergo thematic analysis to identify barriers and facilitators to implementation.

**Discussion:**

This protocol introduces the inaugural study evaluating the feasibility, acceptability, and appropriateness of a low-cost, independently developed AI agent tailored for rural physicians managing older adults with both hypertension and diabetes. It aims to generate empirical data on implementation processes, participant experiences, and contextual barriers. This evidence will directly inform the refinement of recruitment strategies, optimization of intervention protocols and AI agent functionality, and support future large-scale randomized controlled trials (RCTs). The study addresses critical practical challenges in deprescribing within resource-limited rural settings.

**Clinical Trial Registration:** https://www.chictr.org.cn/showproj.html?proj=309897, identifer ChiCTR2600126204.

## 1 Introduction

Hypertension and diabetes are among the most common chronic noncommunicable diseases, and their coexistence is rising worldwide (1). The World Health Organization estimates about 1.13 billion people with hypertension and 537 million with diabetes globally, and the prevalence of hypertension among patients with type 2 diabetes mellitus (T2DM) ranges from 50% to 80% (1–3). Recent national surveys in China report adult prevalences of 27.5% for hypertension and 11.9% for diabetes, and 87.6% of diabetic patients older than 65 have concomitant hypertension (4, 5). The prevalence in rural areas is increasing faster than in urban areas. Primary care physicians in these settings confront multiple challenges, including shortages of medical resources, insufficient healthcare personnel, and limited capacity for chronic disease management. As a result, patients with comorbid hypertension and diabetes frequently require multiple medications, which leads to common polypharmacy and a high prevalence of potentially inappropriate medications (PIMs) (6). These medication-related problems raise the risk of adverse drug reactions, increase medical expenditures, and can result in severe outcomes such as hospitalization and death (6). Reducing polypharmacy and PIMs is therefore an urgent priority for improving care for comorbid hypertension and diabetes in rural regions. The present study is specifically tailored to China's rural primary healthcare context. Its implementation findings may inform similar resource-limited settings internationally, although transferability would require further evaluation.

Deprescribing is the systematic identification and supervised discontinuation of medications that may cause harm or no longer yield clinical benefit, intended to reduce polypharmacy and optimize therapeutic outcomes (7). Recent randomized controlled trials (RCTs) have shown that deprescribing improves patient outcomes. A 2024 review of 17 meta-analyses found that deprescribing reduces inappropriate medication use by 0.8–1.0 medications among patients with polypharmacy, improves Medication Appropriateness Index (MAI) scores by 0.6 points, and lowers all-cause mortality by 26%–38% (8). In addition to reducing adverse drug events and related risks, deprescribing can enhance quality of life and treatment adherence (9). In patients with both hypertension and diabetes, deprescribing may yield further clinical benefits, such as better blood pressure control and greater glycemic stability; for example, stopping unnecessary antihypertensive agents or tailoring glucose-lowering regimens can reduce the risks of hypotension and hypoglycemia while preserving or improving disease control (10).

Despite these benefits, primary care physicians face major obstacles in implementing deprescribing, with insufficient clinical knowledge and a lack of decision-support tools identified as core barriers (11). Additional barriers may include patients' reluctance to discontinue familiar medications and physicians' concerns about adverse consequences and professional responsibility following withdrawal (12). Systematic reviews note that organizational challenges in primary care include the absence of evidence-based guidelines for multimorbidity and of shared clinical decision support systems (CDSS) (12). Although information technology has been investigated to support deprescribing (13, 14), traditional CDSS entail high development and maintenance costs, require deep integration with existing electronic health records, and depend on static clinical knowledge bases (15). Moreover, recommendations from conventional CDSS typically follow standardized rules, which limits their ability to accommodate individualized patient characteristics and the complex clinical contexts of multimorbidity. These constraints impede the widespread deployment and sustainable use of CDSS in resource-limited rural settings.

Recent advances in artificial intelligence (AI) for healthcare have opened new avenues to address the limitations of traditional information systems (16). The rise of large language models (LLMs) has broadened AI's role in clinical decision support (17). As generative deep-learning models, LLMs can interpret and produce human-like natural language and exhibit strong abilities in knowledge synthesis, reasoning, and dialogue (17). Clinicians may describe patient states in plain language, and LLMs can generate context-aware, targeted responses (18). Several studies have examined LLM applications in deprescribing (19–21). For instance, multiple LLMs (e.g., ChatGPT, Gemini, Claude, Llama) have shown satisfactory performance in identifying dosing errors, detecting drug-drug interactions, recommending alternative therapies, and making appropriate management suggestions (22, 23). Nonetheless, conventional LLMs typically operate via single-step interactions that yield static advice, which constrains their utility in complex clinical situations that demand multi-source integration and multi-step reasoning.

To address these limitations, the AI agent has emerged as an evolutionary form of LLM (24). An AI agent can actively retrieve and integrate multi-source heterogeneous data and synthesize patient information across temporal dimensions. It can also dynamically adjust decision paths using iterative reasoning and self-verification. Moreover, an AI agent can interact in real time with external clinical tools, such as guideline knowledge bases, thereby improving decision accuracy and interpretability. By integrating reasoning, planning, memory, and tool use, the AI agent advances from passive response to proactive decision-making. These capabilities make AI agents better suited than traditional LLMs for deprescribing tasks that require assessment of polypharmacy, multimorbidity, and individualized risk factors. A systematic review found that AI agent systems consistently outperform base LLMs in clinical task accuracy, with a median improvement of 53 percentage points (24); the advantage was especially large for discrete tasks such as medication dosing calculations and evidence retrieval.

Previous studies have shown the potential of LLMs and AI agents for medication management, but important gaps remain. Most work consists of small-scale exploratory or retrospective analyses and lacks prospective designs to validate the effectiveness and feasibility of AI agent–assisted interventions. Nearly all published studies were conducted in high-resource settings in developed countries, and few have examined applications in low-resource rural areas of China. Research has emphasized isolated medication decision tasks rather than the comprehensive deprescribing scenarios encountered by older patients with comorbid hypertension and diabetes. As a result, the feasibility and acceptability of AI agent–assisted medication management in routine clinical practice remain unevaluated. Developing an AI agent for clinical medication guidance tailored to rural China and assessing its feasibility, acceptability, and appropriateness in rural practice would therefore have substantial scientific and practical value. The proposed agent integrates an evidence-based knowledge base comprising Chinese guidelines for hypertension management and type 2 diabetes in older adults, the 2023 AGS Beers Criteria, drug-drug interaction resources, and pharmaceutical package inserts to support individualized medication review.

This protocol outlines a feasibility study to evaluate the use of an AI agent by rural Chinese physicians for deprescribing in older patients with comorbid hypertension and diabetes, to assess feasibility, acceptability, and appropriateness, and to identify facilitators and barriers to implementation.

## 2 Methods and analysis

### 2.1 Study design

This protocol outlines a prospective feasibility study. Feasibility studies provide essential preliminary evidence on novel interventions before large-scale RCTs and inform the design of subsequent definitive trials. The study will use quantitative and qualitative methods to assess the feasibility, acceptability, and appropriateness of a proprietary clinical medication-guidance AI agent for helping rural physicians implement deprescribing, and to identify barriers and facilitators to implementation. This protocol follows the SPIRIT 2025 (25) and SPIRIT-AI (26) reporting guidelines.

### 2.2 Study setting

The study will take place in rural primary healthcare institutions across 11 administrative regions of Zhejiang Province, China (Hangzhou, Ningbo, Wenzhou, Jiaxing, Huzhou, Shaoxing, Jinhua, Quzhou, Zhoushan, Taizhou, and Lishui). Each region will recruit 2–3 primary healthcare facilities. Participating institutions must have annual outpatient visits exceeding 5,000 consultations, an electronic medical record system, and internet connectivity infrastructure. Administrative region, participating institution, and relevant contextual information on healthcare resources and digital access will be recorded to support interpretation of variation in tool use and recommendation implementation. Regional patterns will be explored descriptively; formal comparisons of AI performance across regions are not planned.

### 2.3 Participant recruitment

The research team will distribute recruitment notices to eligible rural health clinics in the study regions through local health administrative authorities. Materials will state the study purpose, eligibility criteria, expected time commitment, and participant rights and protections. Interested rural physicians may register voluntarily through designated channels. The research team will screen applicants against predefined inclusion and exclusion criteria. For applicants who pass initial screening, research personnel will contact them individually by telephone or WeChat to explain the study objectives, intervention content, data collection procedures, and potential risks and benefits, and will provide written informed consent documents for review. After confirming willingness to participate and receiving signed consent, rural physicians will be formally enrolled in the study. Enrolled rural physicians will recruit patients by consecutively screening eligible individuals who meet the inclusion and exclusion criteria during routine clinical practice. For eligible patients who express interest, physicians will explain the study purpose, the goals and procedures of deprescribing, data confidentiality measures, and the voluntary nature of participation, and will obtain written informed consent.

Participants may withdraw from the study at any time without providing a reason, and withdrawal will not affect access to subsequent medical care. Research staff will also terminate participation when withdrawal criteria are met in the following situations: (1) participants experience serious adverse events or health deterioration that make continued participation potentially harmful; (2) participants become unable to comply with protocol requirements because of circumstances such as relocation or prolonged absence that prevent completion of follow-up; (3) participants violate key protocol provisions in a way that compromises data integrity or study conduct; (4) research staff determine that termination is in the participant's best interest. All withdrawal cases, whether voluntary or terminated under these criteria, will be documented in detail, including timing, reasons, and the participant's study status at withdrawal, to ensure transparency and accurate data analysis.

### 2.4 Eligibility criteria

Rural physician inclusion criteria: (1) at least two years of clinical practice in rural primary healthcare institutions; (2) possession of a practicing physician qualification; (3) current management of at least 100 patients with concurrent primary hypertension and type 2 diabetes mellitus; (4) demonstrated proficiency in basic computer operations and Internet use. Exclusion criteria: (1) anticipated resignation or extended leave during the study period; (2) concurrent participation in other clinical research that could confound study outcomes; (3) refusal to provide written informed consent.

Patient inclusion criteria: (1) age 65 years or older; (2) documented diagnosis of primary hypertension and type 2 diabetes mellitus according to ICD-10 criteria; (3) currently receiving five or more long-term medications (including prescription and over-the-counter drugs); (4) expected to remain in the local area for the duration of the study and be available for follow-up visits. Exclusion criteria: (1) severe cognitive impairment or psychiatric illness that precludes participation; (2) life expectancy less than 12 months; (3) acute cardiovascular event or hospitalization for acute exacerbation of a chronic disease within the preceding three months; (4) refusal to provide written informed consent.

### 2.5 Sample size

In line with feasibility study methodological guidelines, this protocol does not require a formal statistical sample size calculation. Previous pilot and feasibility studies have used sample sizes of 20–50 participants (27). Given the study's complexity and resource constraints, we plan to recruit approximately 30 rural physicians and about 150 eligible patients under their care.

### 2.6 Outcomes

The primary outcome is the change in the number of long-term medications, defined as prescription drugs used continuously for ≥30 days (excluding as-needed medications), measured by comparing baseline values with those at 6 months post-intervention. A reduction in medication count was selected as the primary outcome because it provides the most direct indication of whether rural physicians can implement deprescribing in routine practice, which is the central question of this feasibility study. This endpoint is also objective, low-burden, and reliably ascertainable from electronic health records, and it is widely reported in deprescribing research, where interventions reduce medication use by 0.8–1.0 medications among patients with polypharmacy, thereby providing a benchmark for interpreting the present findings (8). Medication counts will be obtained from electronic health records and prescription audits and independently verified by the research team to ensure data accuracy.

Secondary outcomes include: (1) number of potentially inappropriate medications (PIMs), assessed using the 2023 Chinese criteria for potentially inappropriate medications in older adults and the Beers Criteria (28); (2) Medication Appropriateness Index (MAI) score, based on 10-item criteria that evaluate each medication, with total scores ranging from 0 to 180 where lower scores indicate more appropriate medication use (29); (3) incidence of adverse drug reactions; (4) blood pressure (systolic and diastolic) and its control rate, defined as the proportion of patients with systolic blood pressure <140 mmHg and diastolic blood pressure <90 mmHg; and (5) fasting plasma glucose (FPG), glycated hemoglobin (HbA1c), and glycemic control rate, defined as the proportion of patients with FPG < 7.0 mmol/L and HbA1c < 7.0%.

Intervention acceptability will be measured using standardized instruments to assess rural physicians' perceptions of the AI agent intervention, specifically the Acceptability of Intervention Measure (AIM), the Intervention Appropriateness Measure (IAM), and the Feasibility of Intervention Measure (FIM) (30). Each scale uses a 5-point Likert response format (1 = completely disagree, 5 = completely agree), with higher scores reflecting greater acceptability, appropriateness, and feasibility. We will also document implementation barriers reported by rural physicians during AI agent use, including technical issues, challenges integrating the tool into workflow, difficulties in patient communication, and any corresponding facilitators.

### 2.7 Study procedures

All participating rural physicians will receive access credentials for the Clinical Medication Guidance AI agent developed by our team. The agent runs on the Coze platform and uses the DeepSeek-V3.2 large language model as its core reasoning engine, integrated with a bespoke evidence-based medicine knowledge base [encompassing Chinese guidelines for hypertension management (31) and type 2 diabetes in older adults (32), the 2023 AGS Beers Criteria, drug–drug interaction databases (28), and pharmaceutical package inserts]. Within the clinical pathway, the AI agent operates as a clinical decision support tool to assist physicians with medication risk assessment at the point of prescribing. It identifies contraindications, performs risk stratification, generates management recommendations, and evaluates adherence to guidelines. The tool is intended to improve the accuracy and safety of medication decisions and to increase guideline-concordant prescribing by healthcare professionals such as rural physicians, general practitioners, and clinical pharmacists. The agent was developed using the DeepSeek large language model as its core reasoning engine (33–35). As the complete agent has not yet undergone external validation in rural primary care, its feasibility, acceptability, and implementation-related performance will be examined in the present study.

The AI agent is available through web and mobile interfaces and features straightforward operational workflows. It is deployed either on hospital servers or via legally compliant online AI development platforms to ensure adherence to medical data privacy and security regulations. Rural physicians enter patient demographics (age, sex, weight), diagnostic information, current medication lists, laboratory results, and adverse drug reaction histories sequentially in response to system prompts to obtain individualized deprescribing recommendations. The generated report delivers four core functions: (1) identification of PIMs by screening the current medication list and specifying the particular concern for each PIM; (2) provision of specific prescription adjustment recommendations, presented in a standardized tabular format; (3) documentation of the evidence-based rationale supporting each recommendation; and (4) generation of individualized management plans, including automated follow-up or referral schedules and monitoring indicator checklists derived from the adjustment protocols to support standardized post-deprescribing management. The agent's outputs supply physicians with a comprehensive medication risk assessment and concrete adjustment recommendations, each accompanied by traceable evidence-based justification. These capabilities help clinicians make safer, guideline-concordant, and individualized prescribing decisions, thereby reducing medication errors and improving overall prescribing quality.

Before deploying the AI agent, participating physicians will complete 4 h of online training on deprescribing principles and procedures. After training, physicians must pass an operational assessment by correctly completing at least 2 simulated cases. The research team will provide online technical support and monitor agent use through system-backend surveillance to ensure intervention fidelity. The study employs a dual-track error-monitoring system that combines passive reporting with active surveillance. First, rural physicians are encouraged to contact the research team if they encounter AI-generated medication recommendations that materially conflict with their clinical judgment or the product monograph. Second, with informed consent, researchers will retrieve each day's AI-agent interaction records from participating physicians and systematically evaluate outputs for factual accuracy, clinical appropriateness, safety, and regulatory compliance. With informed consent, the research team will retrieve locally archived AI-agent interaction records daily. All available records will be reviewed by trained clinical pharmacists, with physician consultation for clinically complex cases and senior clinical adjudication when needed. A standardized error log will document the error type, potential clinical severity, review findings, corrective advice, and subsequent action. Feedback will be provided as soon as practicable, with urgent safety concerns communicated immediately upon identification. Retrieval, review, and notification times will be recorded to characterize review coverage and feedback turnaround. Detected errors will be communicated to physicians without delay. These complementary mechanisms reduce the likelihood of undetected errors and create a closed feedback loop for ongoing optimization of the AI agent's performance. For each enrolled patient, an AI-assisted medication review will be scheduled at baseline, 3 months, and 6 months, with additional consultations as clinically indicated. Adequate scheduled use will be defined as completion of a documented review at each of these three time points, yielding at least three interaction records per patient. Active use will be monitored using backend usage timestamps and locally archived consultation records linked by study codes. Monitoring will capture consultation frequency, timing, completion of required inputs, and generation of medication review reports. Reasons for missed scheduled reviews will be documented.

Rural physicians will enter patients' basic information, disease history, and medication history into the AI agent following the system prompts; the agent then analyzes the input and returns recommendations. Physicians subsequently review those recommendations and, drawing on their clinical judgment and knowledge of each patient's circumstances, decide whether to adopt them. For each actionable AI-generated recommendation, the physician will record whether it was fully adopted, partially adopted, or not adopted. Reasons for partial adoption or non-adoption will be documented, including patient preference, patient-specific clinical considerations, competing treatment priorities, insufficient supporting evidence, or safety concerns. For patients selected for deprescribing, physicians must communicate thoroughly, explaining the rationale for discontinuation or dose adjustment, outlining expected benefits and possible risks, and obtaining informed consent. Physicians then update prescriptions according to the final decision and perform follow-up monitoring guided by either the AI agent's follow-up plan or an individualized protocol devised by the physician. For each deprescribing decision, the physician will document the medication affected, the type of change, the clinical rationale, relevant patient-specific considerations and preferences, and the planned monitoring strategy. This record will reflect the physician's final clinical judgment rather than the AI-generated rationale alone and will be retained in the study documentation. In this study, the AI agent uses a server-side stateless conversation strategy, so the server does not retain historical question-and-answer records; this approach reduces the risk of leakage of patients' sensitive health data. Review of prior AI-generated recommendations relies on a local storage mechanism: after each use, physicians locally cache or manually export the complete question-and-answer content produced by the AI agent on their terminal devices, thereby creating personalized decision-support archives. During follow-up visits or adverse-event investigations, these archived AI recommendations can be retrieved for comparative assessment. The intervention period is 6 months. Assessments will occur at baseline and at 1, 3, and 6 months after intervention initiation, comprising three post-baseline follow-up visits. The 1-month visit will assess medication changes, tolerability, and safety; the 6-month visit will provide the endpoint assessment. Additional clinical monitoring will follow the individualized deprescribing plan. If a scheduled visit is missed, research staff will contact the patient or responsible physician to arrange the earliest feasible reassessment. Contact attempts, reasons for missed visits, and actual assessment dates will be documented, and delayed assessments will be identified in the dataset.

### 2.8 Data collection

#### 2.8.1 Quantitative data

After study commencement, the research team will collect baseline data from all recruited rural physicians and patients. Rural physicians will complete detailed surveys reporting demographic information, educational background, and practice characteristics. Patient baseline data will include disease diagnoses, medication lists, blood pressure values (systolic and diastolic), and glycemic measures (FPG, HbA1c). The team will extract each patient's medical record and medication history for the preceding 6 months from electronic medical record systems. Baseline PIM counts and MAI scores will be calculated by a clinical pharmacist using the medication regimen documented before the first AI-assisted medication review and before any intervention-related medication change. Both measures will be reassessed at the 6-month follow-up using the same assessment criteria. Trained research personnel will collect all baseline data according to standard operating procedures.

Patient outcomes include: (1) medication quality indicators: number of long-term medications, count of PIMs, and MAI scores as assessed by a clinical pharmacist; and (2) clinical control indicators: systolic and diastolic blood pressure values and blood pressure control rate, plus glycemic values and glycemic control rate. All patient clinical data will be extracted from electronic health records and prescription audits and independently verified by the research team to ensure accuracy. Physician outcomes comprise intervention acceptability, measured using the AIM, IAM, and FIM questionnaires to capture rural physicians' perceptions of the AI agent. Each instrument uses a 5-point Likert scale (1 = completely disagree, 5 = completely agree), with higher scores denoting greater acceptability, appropriateness, and feasibility. The research team will also collect process indicators, including physician recruitment rate (the proportion of invited physicians among eligible rural physicians who consent) and physician retention rate (the proportion of baseline physicians who complete the 6-month follow-up). Additional usability indicators encompassing data entry and recommendation generation time, perceived ease of use, technical interruptions, and system availability will be summarized descriptively throughout the intervention. Medication changes during follow-up will be documented using available clinical records and patient or physician reports, including the date, medication, type of change, initiating clinician or patient, and stated reason. Each change will be classified as linked to a documented AI-assisted decision, occurring outside the intervention, or of uncertain origin. All data will be entered into REDCap databases with quality checks. For participants who withdraw, the team will document reasons for withdrawal and collect any available outcome data whenever possible. Implementation resource requirements will be described without formal monetary valuation. Under the current study arrangements, platform access does not incur a study-specific charge, and physicians will use existing personal devices and internet connections. Training attendance and duration, together with the number, nature, and duration of technical support contacts where available, will be recorded. These data will characterize support requirements; formal costing and cost-effectiveness analyses are not planned.

#### 2.8.2 Qualitative data

To understand rural physicians' perceptions of the feasibility and acceptability of an AI agent–assisted deprescribing intervention, and to identify implementation barriers and actual user experiences, all rural physicians will be invited to in-depth interviews after follow-up completion. Interviews will be conducted within 4 weeks after completion of the 6-month follow-up whenever feasible, and the actual interval will be recorded. Where appropriate, locally archived consultation records will be used to prompt discussion of specific experiences. Variation in interview timing may nevertheless introduce recall bias and will be considered when interpreting the findings. Interview guides will follow the updated Consolidated Framework for Implementation Research (CFIR) (36) and probe barriers and facilitators across five domains: physicians' evaluations of AI agent characteristics; effects of the external rural healthcare environment on AI-assisted deprescribing implementation; effects of the internal rural healthcare institution environment on implementation; influence of physician-level characteristics on use of the AI agent for deprescribing; and assessment of the AI-assisted deprescribing implementation process. Trained qualitative researchers will conduct each interview, which will last approximately 45–60 min. Interviews will take place by telephone or video conference, will be audio-recorded with participant consent, and will be transcribed verbatim. Research personnel will keep field notes to document nonverbal cues and preliminary analytic observations.

### 2.9 Data analysis

Descriptive statistics will summarize participants' baseline characteristics, physician recruitment and retention rates, and questionnaire results. Continuous variables will be presented as mean ± standard deviation or median (interquartile range), and categorical variables as frequencies and percentages; 95% confidence intervals will also be reported. Although hypothesis testing is not the primary aim, secondary outcome trends will be examined. Given that each physician manages multiple patients and prescribing behavior may vary across physicians, the analyses will account for the clustering of patients within participating physicians. For continuous outcomes, generalized linear mixed models including a random intercept for physician will assess differences between baseline and the 6-month follow-up. For binary outcomes, McNemar's test will be used. Effect sizes and their 95% confidence intervals will be reported. All statistical tests will be two-sided, with *p* < 0.05 indicating statistical significance. No formal subgroup analyses by physician or patient characteristics are planned because the study is not sized to support reliable subgroup comparisons. Quantitative analyses will be conducted using R software. The counts and proportions of medication changes occurring outside the AI-assisted intervention will be summarized separately. The number and proportion of missing observations will be reported by outcome and assessment time point, together with available reasons and patterns of intermittent missingness or loss to follow-up. No imputation is planned. Mixed models will use available outcome observations, whereas paired binary analyses will include participants with both baseline and 6-month measurements. The number included in each analysis will be reported. Interpretation will acknowledge the assumptions underlying available-data analyses and the potential for bias if missingness is related to unobserved outcomes. Data completeness will also inform procedures for a subsequent definitive trial.

Qualitative analysis in this research will be conducted using Braun and Clarke's inductive thematic analysis method (37). The researchers will utilize MAXQDA 20.4.2 software for efficient data management. To enhance the rigor of the analysis, a team of at least three researchers with expertise in primary care and qualitative research methods will be involved in the analysis process. The analysis will follow the six-step framework: (1) familiarize with the data through repeated readings of interview transcripts; (2) perform line-by-line open coding to extract semantic features; (3) consolidate codes into preliminary themes; (4) review and refine themes through team discussion to ensure internal consistency and overall representativeness; (5) define and name each theme; and (6) produce analytical reports that integrate representative quotations with relevant literature.

### 2.10 Patient and public involvement

This study prioritized patient and public participation. During the design phase, the research team invited three rural physician representatives to join discussions, and they offered input on study acceptability, informed consent readability, questionnaire appropriateness, and intervention feasibility. The team incorporated these suggestions into the final protocol after in-depth discussion. Patient and public contributions will be acknowledged explicitly in study publications.

### 2.11 Ethics and dissemination

This protocol was approved by the Ethics Committee of the Second Affiliated Hospital, Zhejiang University School of Medicine [Ethics Review No. (2025) 1983]. The study will be conducted in accordance with the ethical principles of the Declaration of Helsinki 2025 (38). Prior to enrollment, all potential participants, including rural physicians and patients, will receive detailed written and verbal information about the study. Informed consent documents will describe the study purpose, interventions, potential benefits and risks, data confidentiality measures, the voluntary nature of participation, and the right to withdraw at any time. Research staff will confirm that participants understand the information and will obtain written informed consent on a voluntary basis.

The study will implement strict data management and privacy protections to maintain the confidentiality and security of participant information. All collected personal data will be de-identified by replacing names and other identifiers with unique research codes. Electronic records will reside on encrypted, password-protected servers accessible only to authorized research staff. Paper records will be kept in locked file cabinets. Data transmission will use secure, encrypted channels. After study completion, original data will be retained or destroyed in accordance with institutional policies and ethics committee requirements. Participants may request access to, correction of, or withdrawal of their personal data at any stage of the research. If significant protocol modifications become necessary during implementation, the research team will submit amendments to the ethics committee promptly and implement changes only after approval. All protocol revisions will be updated on the clinical trial registration platform and described in the final published reports, including revision content, rationale, and potential effects on the study. Major revisions will also be communicated promptly to enrolled participants, and informed consent will be re-obtained when required.

A comprehensive adverse event monitoring and reporting system will be maintained throughout the study. The research team will closely monitor all intervention-related adverse events, defined as any medical occurrences that harm patient health during the study, and serious adverse events, defined as events that cause death, are life-threatening, require hospitalization, result in persistent disability, or constitute other significant medical conditions. Each adverse event will be classified and recorded by severity and by its relationship to the intervention. Serious adverse events, including those that cause hospitalization, are life-threatening, or result in death, will be reported to the ethics committee within 24 h of identification. If unacceptable safety risks attributable to the intervention are identified, the study may be terminated prematurely. The incidence, severity, and management of all adverse events will be reported transparently in the study results.

This study protocol has been prospectively registered with the Chinese Clinical Trial Registry (ChiCTR) (ChiCTR2600126204). Study implementation will follow the SPIRIT 2025 (25) and SPIRIT-AI (26) reporting requirements, and results publication will adhere to the CONSORT 2025 (39) and CONSORT-AI (40) extensions for pilot and feasibility studies. The research team will publicly disseminate findings regardless of outcome to minimize publication bias. If the study is terminated prematurely, the reasons for termination will be reported on the registration platform and in subsequent publications. Study results will be prepared as academic manuscripts for submission to high-quality international peer-reviewed journals, and will be made open access to enhance accessibility.

## 3 Discussion

The comorbidity of hypertension and diabetes is increasingly common in rural areas, where patients often face polypharmacy that raises the risk of adverse drug reactions, reduces adherence, and increases the medical burden (41, 42). Deprescribing is an evidence-based strategy that reduces inappropriate medication use and improves outcomes (43). Conventional deprescribing models, including pharmacist-led medication reviews, multidisciplinary care, clinician education, and computerized decision support (7, 8), generally require adequate staffing, prolonged consultation time, and advanced technical infrastructure, which are often inaccessible in rural primary care settings (12, 15). Consequently, these established approaches have limited rural applicability. The proposed AI agent is designed to complement existing deprescribing frameworks by delivering structured, evidence-based recommendations during routine clinical encounters while retaining clinician autonomy for final decision-making. Whether this tool can alleviate clinical workload and lower resource demands compared with traditional strategies remains unclear. Rural clinicians face multiple barriers to deprescribing, including gaps in clinical knowledge, lack of systematic decision support, and constrained time and resources (44, 45). Although rapid advances in AI present new opportunities, AI-agent–based deprescribing interventions for rural settings are still nascent; their feasibility, acceptability, and implementation barriers remain unevaluated. Thus, this feasibility study is timely and important, as it can provide essential evidence about the potential of AI-assisted interventions in resource-limited environments.

The principal contribution of this protocol is the prospective evaluation of an independently developed AI agent to support rural physicians managing older adults with comorbid hypertension and diabetes. The agent's development and evaluation study remains unpublished, and published validation evidence for this specific system in rural primary care is not currently available. Assessments of the underlying language model cannot establish the performance of the complete agent. The present study will therefore provide prospective implementation evidence and monitored observations of recommendation quality and safety, rather than definitive evidence of clinical effectiveness. The agent is low-cost, easy to operate, and remotely accessible, making it well suited to rural primary healthcare settings (46). Unlike conventional clinical decision support systems, the AI agent requires no complex system integration or costly hardware and software investments; rural clinicians can receive individualized medication-optimization recommendations via the internet. The implementation strategy centers on the AI agent as the primary intervention and includes targeted training and ongoing support. This approach promotes standardized delivery while accommodating the operational constraints of rural medical practice. Training is framed as a supplementary measure rather than the primary intervention to avoid reliance on intensive training resources and thus improve scalability and sustainability. Successful adoption of novel AI technologies depends not only on tool quality but also on implementation strategies that address practical barriers. Accordingly, monthly workshops are included as a core implementation strategy to provide a supportive forum for peer learning and experience exchange, helping rural physicians surmount technical hurdles and practical challenges associated with the AI agent and enabling effective, sustained use in primary care settings (47). The agent can be accessed remotely via existing devices without extra hardware purchases or local electronic health record integration under this study, yet routine deployment demands clinician time, training, technical support and sustained platform and network resources. Since resource consumption will not be monetized, this study cannot confirm large-scale affordability or cost-effectiveness; these resource data will instead inform future economic evaluations of routine deployment support needs and recurrent costs. Long-term sustainability will depend on continued platform access, reliable connectivity, maintenance of clinical knowledge resources, and integration into routine workflows. Subsequent evaluations should determine whether training and technical support can be maintained outside the research setting and characterize the recurrent resources required for wider implementation. Potential unintended consequences include inappropriate medication withdrawal, clinical deterioration, and overreliance on AI recommendations (15). Clinician-led final decisions, documented clinical reasoning, scheduled follow-up, and active review of AI outputs are intended to reduce and detect these risks, although they cannot eliminate them.

The prospective feasibility study design follows recommendations from intervention development and evaluation frameworks and functions as a necessary precursor to a full-scale RCT (48). The primary objective of a feasibility study is to assess implementation and acceptability of an intervention within the target setting rather than to establish clinical efficacy (48). By systematically examining key indicators, including recruitment and retention rates, intervention fidelity, and completeness of data collection, this study will identify potential implementation barriers and inform protocol optimization. Integrating quantitative and qualitative data creates a robust basis for comprehensively evaluating feasibility: quantitative measures supply objective indicators, while qualitative interviews permit in-depth exploration of participants' experiences, attitudes, and suggestions (49). Although the proposed sample size is relatively small, it meets methodological standards for feasibility studies and yields sufficient information to assess critical parameters. Crucially, the study will deliver the methodological groundwork needed for future large-scale RCTs, including optimized recruitment strategies, a refined intervention protocol, selection of appropriate outcome measures, and parameter estimates for sample size calculations, all of which are essential to ensure scientific rigor and efficient use of resources in subsequent research.

This feasibility study is expected to contribute across several domains. Practically, it will provide the first empirical data on the feasibility and acceptability of AI-assisted deprescribing interventions in rural primary care, offering direct guidance for technical implementation and resource allocation. Methodologically, it will lay the groundwork for future rigorous RCTs by validating study procedures, refining the intervention, and identifying critical success factors. At the policy level, the results will supply evidence to support the adoption of digital health technologies, capacity building for rural physicians, and the optimization of chronic disease management services in rural areas. Based on these findings, subsequent research may include multi-center cluster RCTs to rigorously assess the long-term effects of AI-assisted interventions on prescription quality, patient health outcomes, and cost-effectiveness. The lessons learned may also be applied to medication management for other chronic disease comorbidities, thereby promoting responsible AI innovation in primary care. Potential equity benefits must be considered alongside differences in connectivity, device availability, and physicians' digital literacy. Physician-facing access, structured training, and technical support may reduce some barriers without requiring patients to operate the tool themselves. However, the eligibility requirements favor digitally equipped practices, and applicability to the most digitally disadvantaged settings will require further evaluation. Overall, this study explores the integration of advanced digital technology with primary care needs and aims to improve medication management and health equity for rural patients with chronic disease.

The study has several inherent limitations, but the research team will employ strategies to mitigate their effects. First, the lack of a control group prevents ruling out confounding influences on outcomes; however, this limitation is consistent with the aims of a feasibility study, which focuses on assessing implementation rather than establishing causality. Clinical outcome data will therefore be treated as secondary, exploratory measures. Second, as the study is restricted to specific regions of an eastern Chinese province, its findings have limited generalizability. While rural sites spanning varied economic and healthcare resource levels will be recruited to boost representativeness, cross-setting differences in clinical case mix, input data completeness, digital infrastructure and prescribing practices may affect AI-agent performance and implementation, precluding direct extrapolation beyond this province, though these diverse sites can help identify contextual factors for later external evaluation. Third, the 6-month follow-up may be insufficient to capture the long-term safety and health consequences of deprescribing; nevertheless, this interval is reasonable for a feasibility assessment. Subsequent randomized controlled trials will adopt longer follow-up periods to evaluate long-term effects. Fourth, using convenience sampling for participant recruitment may introduce selection bias, since participating physicians might already be inclined toward deprescribing or novel technologies. Fifth, the pre-intervention training and physicians' awareness of being monitored may influence prescribing behavior independently of the AI agent itself. This potential effect is inherent to unblinded feasibility studies and does not compromise the feasibility-oriented objectives of the study, and the semi-structured interviews will further explore and interpret its influence. Sixth, the lack of patient-reported outcomes restricts direct evaluation of patients' experiences and understanding of deprescribing, yet this study yields preliminary evidence on physician-focused implementation and clinical monitoring to inform future evaluations with adapted, tested patient-reported measures. This approach is acceptable at the feasibility stage because it permits evaluation of the intervention under relatively favorable conditions.
